# High-Throughput Transcriptomic Profiling Reveals the Inhibitory Effect of Hydroquinine on Virulence Factors in *Pseudomonas aeruginosa*

**DOI:** 10.3390/antibiotics11101436

**Published:** 2022-10-19

**Authors:** Nontaporn Rattanachak, Sattaporn Weawsiangsang, Krai Daowtak, Yordhathai Thongsri, Sukunya Ross, Gareth Ross, Nungruthai Nilsri, Robert A. Baldock, Sutatip Pongcharoen, Touchkanin Jongjitvimol, Jirapas Jongjitwimol

**Affiliations:** 1 Biomedical Sciences Program, Faculty of Allied Health Sciences, Naresuan University, Phitsanulok 65000, Thailand; 2 Department of Medical Technology, Faculty of Allied Health Sciences, Naresuan University, Phitsanulok 65000, Thailand; 3 Cellular and Molecular Immunology Research Unit, Faculty of Allied Health Sciences, Naresuan University, Phitsanulok 65000, Thailand; 4 Department of Chemistry, Faculty of Science, Naresuan University, Phitsanulok 65000, Thailand; 5 Centre of Excellence in Biomaterials, Faculty of Science, Naresuan University, Phitsanulok 65000, Thailand; 6 School of Pharmacy and Biomedical Sciences, Faculty of Science and Health, University of Portsmouth, Portsmouth PO1 2DT, UK; 7 Division of Immunology, Department of Medicine, Faculty of Medicine, Naresuan University, Phitsanulok 65000, Thailand; 8 Biology Program, Faculty of Science and Technology, Pibulsongkram Rajabhat University, Phitsanulok 65000, Thailand

**Keywords:** anti-virulence factor, biofilm, flagellar assembly, hydroquinine, pyocyanin, *Pseudomonas aeruginosa*, transcriptomic analysis

## Abstract

Hydroquinine is an organic alkaloid compound that exhibits antimicrobial activity against several bacterial strains including strains of both drug-sensitive and multidrug-resistant *P.* *aeruginosa*. Despite this, the effects of hydroquinine on virulence factors in *P. aeruginosa* have not yet been characterized. We therefore aimed to uncover the mechanism of *P. aeruginosa* hydroquinine-sensitivity using high-throughput transcriptomic analysis. We further confirmed whether hydroquinine inhibits specific virulence factors using RT-qPCR and phenotypic analysis. At half the minimum inhibitory concentration (MIC) of hydroquinine (1.250 mg/mL), 254 genes were differentially expressed (97 downregulated and 157 upregulated). We found that flagellar-related genes were downregulated by between −2.93 and −2.18 Log_2_-fold change. These genes were consistent with the analysis of gene ontology and KEGG pathway. Further validation by RT-qPCR showed that hydroquinine significantly suppressed expression of the flagellar-related genes. By analyzing cellular phenotypes, *P. aeruginosa* treated with ½MIC of hydroquinine exhibited inhibition of motility (30–54% reduction) and pyocyanin production (~25–27% reduction) and impaired biofilm formation (~57–87% reduction). These findings suggest that hydroquinine possesses anti-virulence factors, through diminishing flagellar, pyocyanin and biofilm formation.

## 1. Introduction

Infectious diseases caused by drug-resistant pathogens are one of the most pressing problems facing public health in many countries, leading to more difficult-to-treat microbial infections [1]. These pathogens are a significant cause of human mortality, especially infections with multidrug-resistant (MDR) pathogens [2]. *Pseudomonas aeruginosa* is one of the major MDR bacteria isolated from patients with nosocomial infections [3], e.g., bacteremia [4], pneumonia [5], urinary tract infections [6], and post-surgical infections [7,8]. Importantly, nosocomial infections by MDR *P. aeruginosa* cause mortality in 15–61% of cases in different countries [9,10,11,12]. Antimicrobial resistance can occur through several pathways including the production of enzymes, decreasing outer membrane permeability, overproducing efflux pumps [13], or alternatively, inducing virulence factor production [14]. In the case of *P. aeruginosa*, the bacterium produces certain virulence factors that facilitate colonization of the host cells, damage tissue, and help the pathogen evade the host defenses [15,16]. Mechanistically, there are several factors in *P. aeruginosa* that aid virulence, including flagella, pili, degrading enzymes, biofilm, toxins, specialized secretion systems, pigments (e.g., pyocyanin), and cell-to-cell signaling molecules [15,16,17,18].

A novel approach in treating bacterial infections is to specifically block the virulence factors instead of killing and/or inhibiting bacterial growth directly [19]. The strategies for inhibiting bacterial virulence include inhibiting bacterial adhesion, toxin production, bacterial secretory systems as well as downregulation of virulence genes and blocking of cell-to-cell signaling [19]. Importantly, some naturally occurring compounds possess antimicrobial properties [20,21]. Critically, numerous agents have exhibited anti-virulence factors (e.g., anti-biofilm formation, anti-quorum sensing (QS) signaling, anti-motility, anti-pigment production) [22,23,24,25,26,27] against various pathogens including *P. aeruginosa*, *Escherichia coli*, *Staphylococcus aureus* [16,19].

Hydroquinine, an alkaloid organic compound found in particular natural products [28,29,30], was recently reported to inhibit and kill drug-sensitive (DS) and MDR strains of *P. aeruginosa* [31]. In addition, other alkaloid compounds have been reported to possess antimicrobial properties and anti-virulence factors in both Gram-positive and Gram-negative bacteria, such as inhibition of QS signaling and pigment production in *P. aeruginosa* [26], inhibiting biofilm formation in *E. coli* and *P. aeruginosa* [24] and inhibiting virulence gene expression in *S. aureus* [32]. However, the inhibitory effect of hydroquinine on virulence factors in *P. aeruginosa* has not yet been investigated. We, therefore, aimed to investigate the mechanism of action of hydroquinine against *P. aeruginosa* using high-throughput transcriptomic analysis. We also investigated whether hydroquinine could inhibit virulence factors in *P. aeruginosa.* Here, we demonstrate that several flagella-related genes are downregulated in hydroquinine-treated *P. aeruginosa*. Furthermore, we show that hydroquinine reduces motility, gene expression of the *rhlI/R* QS system, pyocyanin production and biofilm formation in both DS and MDR strains of *P. aeruginosa*.

## 2. Materials and Methods

### 2.1. Bacterial Strains and Cultivation

The bacterial reference strains used in this study were obtained from American Type Culture Collection (ATCC), namely *P. aeruginosa* ATCC 27853 (drug sensitive; DS) and *P. aeruginosa* ATCC BAA-2108 (multidrug resistant strain; MDR). All bacterial samples were grown in tryptone soya agar (TSA; Cat. No. CM0131, Oxoid, Basingstoke, UK) at 35 ± 2 °C for 18–24 h [31]. For inoculum preparation, the inoculum was adjusted in exponential growth phases to achieve turbidity equivalent to a 0.5 McFarland standard (1–2 × 10^8^ CFU/mL) before being used in each experiment. For the experiment of hydroquinine treatment, *P. aeruginosa* was cultured with or without the corresponding concentration of hydroquinine in Mueller–Hinton broth (MHB; Cat. No. CM0405B, Oxoid, Basingstoke, UK) at 35 ± 2 °C for a certain period as designed in each experiment.

### 2.2. Hydroquinine Preparation

Hydroquinine was purchased from Sigma-Aldrich (Cat. No. 522-66-7). Fresh hydroquinine was made before use by dissolving in 50% DMSO and Tween 80 (1:1) solution. The hydroquinine solution was syringe-filtered (through a 0.2 μm pore size) and kept away from the light source [31].

### 2.3. RNA Extraction 

RNA extraction was performed using RNeasy Mini Kit (Cat. No. 74004, QIAGEN, Hilden, Germany) as previously described by Rattanachak et al. (2022) [31]. Total RNA samples were also isolated using RNase-Free DNase Set (Cat. No. 79254, QIAGEN, Hilden, Germany) to remove genomic DNA contamination. For all downstream applications, total RNA samples extracted were quantified and analyzed for purity using a Colibri Microvolume Spectrophotometer (Titertek Berthold, Pforzheim, Germany). The purity of total RNA (A_260_/A_280_) was around 2.0. All RNA extracts were kept at −80 °C for further analysis.

### 2.4. Transcriptomic Analysis and Differential Expression Gene (DEG) Analysis 

The exponential phase cultures of *P. aeruginosa* ATCC 27853 were grown in 30 mL of MHB and treated with either half MIC of hydroquinine (1.250 mg/mL) or DMSO and Tween 80 as an untreated control. Each culture was incubated at 35 ± 2 °C for 1 h, with shaking at 200 rpm. The bacterial pellets were collected by centrifugation at 5000 rpm and 4 °C for 10 min [31]. The total RNA from each sample was then extracted. The RNA sequencing and transcriptomic analysis was performed by Macrogen Inc. (Seoul, South Korea). Briefly, the total RNA integrity was performed using Agilent 2100 Bioanalyzer (Agilent, Santa Clara, CA, USA) with an RNA integrity number (RIN) greater than or equal to 7. The TruSeq stranded total RNA kit (Illumina, San Diego, CA, USA) was used for library preparation. The cDNA library was prepared by cDNA random fragmentation, followed by 5’ and 3’ adapter ligation. Adapter-ligated fragments were then PCR amplified and gel purified. The cDNA library was sequenced on a flow cell using high-throughput 2 × 150 nt, pair-end mode on an Illumina HisSeq 2100 platform (Illumina, San Diego, CA, USA). The RNA sequencing analysis included a quality check for raw sequencing data, read mapping, expression quantification, differential expression genes (DEGs) analysis, and function enrichment analysis as previously described [31]. Briefly, the passed filter reads were mapped onto *Pseudomonas aeruginosa* PAO1 genome reference using Bowtie2 [33]. Transcript quantification and DEGs analysis were then conducted using Feature-count and edgeR, respectively [34]. The results of DEGs were summarized using the significant criteria of −2 ≥ log_2_ fold change ≥ 2 and a false discovery rate (FDR) ≤ 0.05. The RNA sequencing analysis generated 4,465,440 and 3,665,572 raw sequencing reads for hydroquinine treated and untreated conditions, respectively. Total read bases were approx. 1.35 × 10^9^ and 1.11 × 10^9^ bp, respectively, with a great Phred quality score (Q20) of greater than 97.97%. After mapping to the genome of *P. aeruginosa* (PAO1 genome reference), the read counts of functional genes assigned features in treated and untreated with hydroquinine were 3,565,444 (80.6%) and 2,968,484 (81.7%), respectively. Functional annotation of the significant DEGs in Gene ontology (GO) terms and KEGG pathway were also analyzed using the Database for Annotation, Visualization, and Integrated Discovery (DAVID) (https://david.ncifcrf.gov/ 2021 updated database, accessed on 20 September 2022).

### 2.5. Complementary DNA (cDNA) Synthesis and Quantitative Reverse Transcription Polymerase Chain Reaction (qRT-PCR)

Before cDNA synthesis, the RNA samples may contain genomic DNA residues; thus, they were removed as previously described [31]. The cDNA synthesis was performed using a FIREScript RT cDNA synthesis kit (Cat. No. 06-15-00050, Solis Biodyne, Tartu, Estonia), by following the manufacturer’s instruction. Briefly, 2 µL of 10×Reverse Transcription buffer, 500 ng of RNA, 1 µL of reverse transcriptase, 1 µL of 100 µM oligo (dT) primers, 0.5 µL of dNTP Mix, 0.5 µL of 40 U/µL RNase inhibitor and RNase-free water up to 20 µL final volume were added to the reaction tube. The annealing step was performed at 25 °C for 5 min, and the reverse-transcription step was performed at 45 °C for 30 min, followed by enzyme inactivation at 85 °C for 5 min. The concentration of cDNA synthesized was measured prior to downstream analysis.

For qRT-PCR, in both DS and MDR *P. aeruginosa* samples, the experiments were performed using HOT FIREPol^®^ EvaGreen^®^ qPCR Mix Plus (Cat. No. 08-25-00001, Solis Biodyne, Tartu, Estonia) by following the manufacturer’s instructions. The cDNA synthesized was then used as a PCR template. The specific primers for each gene and their associated annealing temperatures are shown in Table 1. The *16S rRNA* of *P. aeruginosa* was used as a reference gene. The qRT-PCR cycling conditions were as follows: 40 cycles of denaturation at 95 °C for 15 s, proper annealing step ranging at 56.0–58.5 °C for 20 s, and extension at 72 °C for 20 s. The *16S rRNA* gene was used as a housekeeping reference to calculate the relative expression levels of the genes using a 2^−ΔΔCt^ method. 

### 2.6. Motility Assays

The motility of both *P. aeruginosa* strains was evaluated using swimming and swarming motility assays with the following modifications [35]. Briefly, Luria–Bertani (LB) solidified with 0.3% of agarose was used to investigate swimming motility, and LB with 0.5% agarose and D-glucose was used for swarming motility. Both assays were performed in 6-well plates (35 mm diameter) containing 3 mL of corresponding media with hydroquinine at concentrations of 2.500, 1.250 and 0.625 mg/mL for the DS strain as well as of 1.250, 0.625 and 0.312 mg/mL for the MDR strain, according to the MIC values reported in Rattanachak et al. (2022) [31]. For the swimming motility, the assay was performed by strapping the inoculum in the center of the agar thickness. For the swarming motility, 2 µL of the inoculum was pipetted on the central agar. The diameter zones of the swimming and swarming motilities were measured after incubation at 35 ± 2 °C for 24 h.

### 2.7. Detection of Quorum Sensing (QS) Signaling-Related Virulence Factors

In this study, specific quorum sensing (QS) signaling-related virulence factors were investigated in both DS and MDR *P. aeruginosa* strains after treatment with and without hydroquinine at different concentrations. The experiments were divided into three main aspects. Firstly, the *rhlI* and *rhlR* transcripts, the QS signaling mRNAs, were determined using qRT-PCR as mentioned above. The RNA samples were isolated in both the DS and MDR *P. aeruginosa* strains after the 1 h treatment with ½ × MIC hydroquinine at 1.250 and 0.625 mg/mL, respectively, as well as without hydroquinine as an untreated control. The primers are shown in Table 1. The relative expression levels of the genes were calculated as above using the 2^−ΔΔCt^ method. 

Secondly, pyocyanin production was determined using colorimetric spectrometry [35]. Briefly, MHB-based supernatants of each inoculum after treatment with and without a particular hydroquinine concentration at 35 ± 2 °C for 24 h were separated by centrifugation at 4000 rpm for 15 min. The supernatant was then syringe-filtered (a 0.2 μm in pore size filter). The pyocyanin pigment extraction was performed using chloroform at a ratio of 2:3 and re-extracted with 1.0 mL of 0.2 M HCl. MHB with the corresponding inoculum was the untreated control. The pyocyanin pigment was measured at 540 nm using a microplate reader (PerkinElmer, Waltham, MA, USA), then calculated and reported as percentage of pyocyanin production inhibition.

Lastly, the biofilm mass formation was determined using crystal violet retention assay with the following modifications [36,37]. Briefly, the experiments were performed in 96-well plates. In wells, MHB (200 µL) containing hydroquinine at the final concentrations of 2.500, 1.250, and 0.625 mg/mL with 10% DS inoculum as well as of 1.250, 0.625 and 0.312 mg/mL with 10% MDR inoculum. DMSO and Tween 80 were used as a vehicle control in MHB with the corresponding inoculum as an untreated control. The plates were incubated at 35 ± 2 °C for 24 h. The planktonic cells were carefully removed and washed with sterile distilled water three times. The biofilm mass in each well was then dried at 60 °C for 45 min. The adherent biofilm cells were stained with 0.1% (*w*/*v*) crystal violet for 20 min at room temperature. The crystal violet was washed with sterile distilled water three times, and then re-dissolved with 95% ethanol (*v*/*v*). The optical density was measured at 595 nm using a microplate reader (PerkinElmer, Waltham, MA, USA) and then calculated as percentage of biofilm formation inhibition. 

### 2.8. Statistical Analysis

All the experiments were performed independently in triplicate. Where appropriate, results are shown as means ± standard deviation (SD). An independent student t test using IBM SPSS statistics version 23 (Armonk, NY, USA) was used to test statistically significant mean differences between both comparing treated and untreated groups and comparing treated and vehicle control groups. GraphPad Prism version 8.2.0 (San Diego, CA, USA) was created and analyzed all graphs. For all analyses, significant differences were reported for *p* values < 0.05.

## 3. Results

### 3.1. Effects of Hydroquinine on the Transcriptomic Profile of P. aeruginosa ATCC 27853

**Figure 1:**
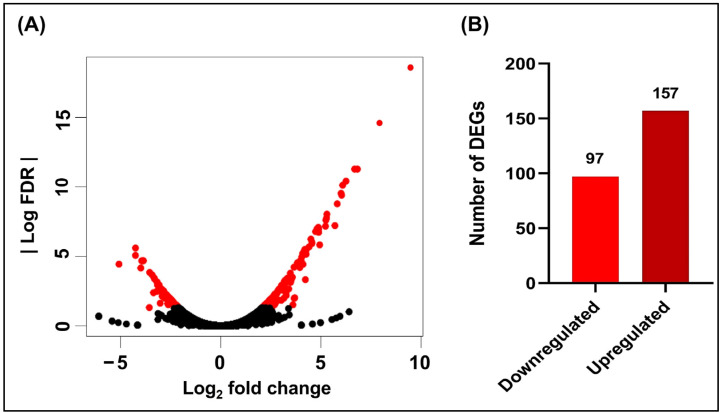
The differential expressed genes (DEGs) of *P. aeruginosa* ATCC 27853 in response to hydroquinine, showing (**A**) volcano plot with the statistically significant DEGs as red dots and non-significant DEGs as black dots as well as (**B**) the DEG number of downregulation and upregulation.

RNA sequencing experiments were performed to investigate the effect of hydroquinine on the global gene expression. *P. aeruginosa* ATCC 27853 was planktonically grown with the exposure to 1.250 mg/mL of hydroquinine for 1 h and compared to the untreated control. From DEGs analysis, 254 genes were found to be differentially expressed in hydroquinine-treated cells, compared to the untreated control as illustrated in Figure 1. Of the 254 genes, 157 genes (log_2_-fold change from 2.09 to 9.47) were upregulated and 97 genes (log_2_-fold change from −5.07 to −2.09) were downregulated.

**Figure 2:**
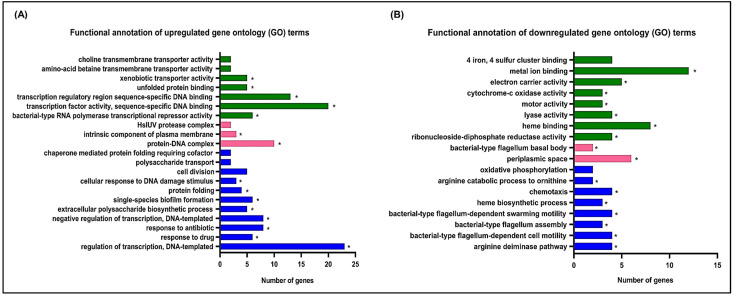
Enriched gene ontology (GO) terms of (**A**) significantly upregulated DEGs and (**B**) significantly downregulated DEGs after *P. aeruginosa* ATCC 27853 treated with hydroquinine for 1 h. The blue bars represent biological processes, the pink bars represent cellular components, and the green bars represent molecular functions. The asterisk (*) symbol is *p* < 0.05 as considered significant.

**Figure 3:**
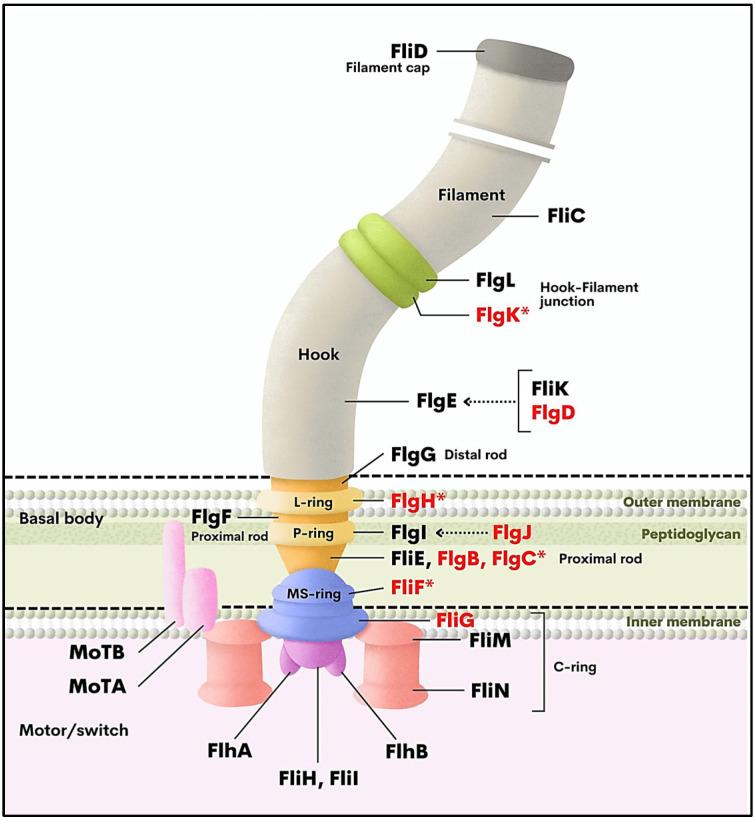
The flagellar structure of *P. aeruginosa* and gene products involved in flagellar assembly and/or regulation. The red labels represent the downregulated DEGs of *P. aeruginosa* in response to hydroquinine for 1 h, according to the investigation by transcriptome analysis. The asterisk (*) symbol represents the genes selected for reverifying the expressional accuracy using qRT-PCR. The diagram was modified from Nolan et al. (2018) [38].

The DAVID online database was used to perform the gene ontology (GO) analysis to investigate the functions of DEGs. The 254 DEGs represented several biological processes (BP), cellular components (CC) and molecular functions (MF), which are ordered by enrichment scores (Figure 2). The upregulated gene groups are annotated and shown in Figure 2A when *P. aeruginosa* ATCC 27853 was exposed to 1.250 mg/mL of hydroquinine. Regulation of DNA-templated transcription was the most significantly enriched BP GO-term. For CC, protein–DNA complex had the highest number of upregulated genes. For the MF, a number of genes involved in the transcription factor activity of sequence-specific DNA binding was most enriched. The downregulated DEG functions in the hydroquinine exposure are displayed in Figure 2B. Functional annotation shows that downregulated DEGs are associated with flagella-related functions. This was consistent across GO categories (BP, MF and CC). Moreover, using the KEGG pathway analysis, genes associated with the flagella assembly mechanism were also affected in response to hydroquinine in *P. aeruginosa* ATCC 27853. The relative fold change of the DEGs involved in flagella assembly showed significant downregulation (log_2_-fold change from −2.93 to −2.18) (Table 2 and Table S1). The flagella-related genes are involved in the flagella hook–filament junction (*flgK*), flagella hook modification protein (*flgD*), flagella basal body (*flgB*, *flgC*, *flgH*, *flgJ*, *fliF*), and flagella motor (*fliG*) (Figure 3). 

### 3.2. Validation of Downregulation of Flagella-Related Genes by qRT-PCR

**Figure 4:**
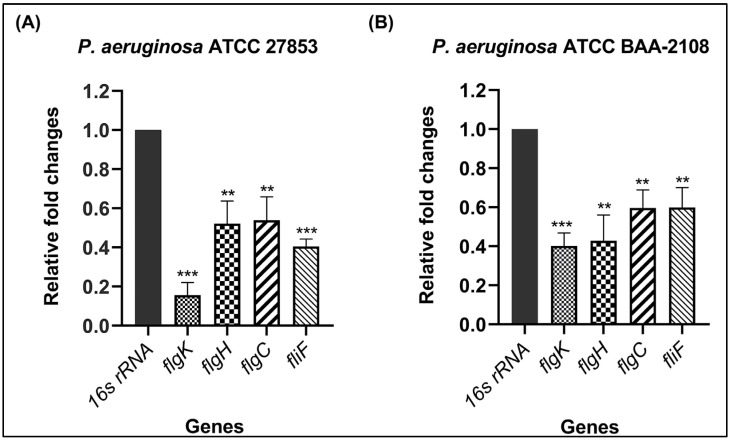
The relative expression of flagellar assembly genes in (**A**) *P. aeruginosa* ATCC 27853 strain with hydroquinine at 1.250 mg/mL for 1 h and (**B**) *P. aeruginosa* ATCC BAA-2108 strain treated with hydroquinine at 0.625 mg/mL for 1 h, compared to the corresponding untreated control. The asterisk ** and *** symbols were *p* < 0.01 and *p* < 0.001, respectively.

Four representative genes (*flgK*, *flgH*, *flgC* and *fliF*) involved in constructing the flagellar assembly were selected to validate the transcriptomic results. The relative gene expression levels of the four genes were analyzed by qRT-PCR in both the DS and MDR *P. aeruginosa* strains treated either with or without 1.250 and 0.625 mg/mL of hydroquinine for 1 h, respectively. As shown in Figure 4, the relative gene expression of all of the flagellar genes tested in both the DS and MDR strains showed downregulation in response to hydroquinine. Specifically, in the DS *P. aeruginosa* strain, the relative expression levels of *flgK* were significantly decreased by 0.15 ± 0.06-fold. The genes involved in flagellar basal body, *flgH*, *flgC*, and *fliF* showed that the relative expression levels were significantly decreased 0.52 ± 0.11, 0.53 ± 0.12, and 0.40 ± 0.03-fold, respectively (Figure 4A). For the MDR *P. aeruginosa* ATCC BAA-2108, after exposure to 0.625 mg/mL of hydroquinine for 1 h, the relative expression levels of the flagella hook-associated gene (*flgK*) significantly decreased at 0.40 ± 0.06-fold. The relative expression levels of the genes involved in the flagellar basal body, *flgH*, *flgC*, and *fliF* exhibited significant repression of 0.42 ± 0.13, 0.59 ± 0.09 and 0.59 ± 0.10-fold, respectively (Figure 4B). The results revealed that hydroquinine negatively affects the expression of genes involved in generating the flagellar assembly of both DS and MDR *P. aeruginosa* strains.

### 3.3. Hydroquinine Disrupts Motility in Both P. aeruginosa Strains

**Figure 5:**
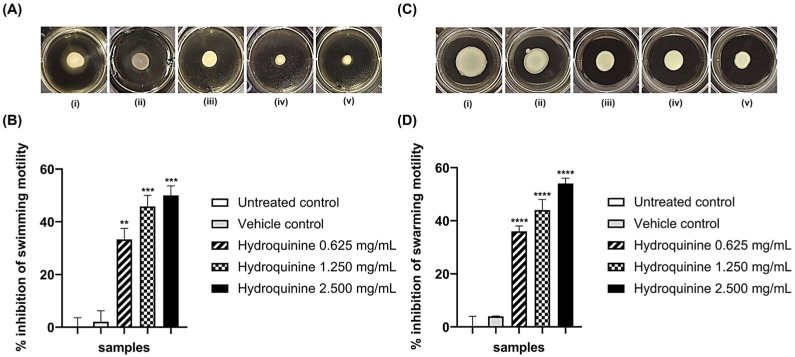
Anti-motility effects of different concentrations of hydroquinine on (**A**,**B**) swimming and (**C**,**D**) swarming patterns in the DS *P. aeruginosa* ATCC 27853 at 35 ± 2 °C for 24 h, labeled (i) untreated controls; (ii) vehicle controls; (iii–v) the hydroquinine concentrations at 0.625, 1.250 and 2.500 mg/mL, respectively. The percentage inhibition of (**B**) swimming and (**D**) swarming motilities by different concentrations of hydroquinine, compared with the control groups. Mean and standard deviation values from triplicate independent are shown. The asterisk **, *** and **** symbols are *p* < 0.01, *p* < 0.001, and *p* < 0.0001, respectively.

**Figure 6:**
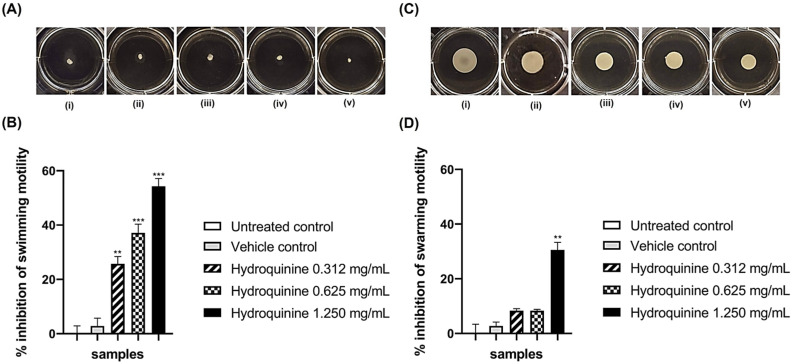
Anti-motility effects of the different concentrations of hydroquinine on (**A**,**B**) swimming and (**C**,**D**) swarming patterns in the MDR *P. aeruginosa* ATCC BAA-2108 at 35 ± 2 °C for 24 h, labeled (i) untreated controls; (ii) vehicle controls; (iii–v) hydroquinine concentrations at 0.312, 0.625 and 1.250 mg/mL, respectively. The percentage inhibition of (**B**) swimming and (**D**) swarming motilities by different concentrations of hydroquinine, compared with the control groups. Mean and standard deviation values from triplicate independent are shown. The asterisk ** and *** symbols are *p* < 0.01 and *p* < 0.001, respectively.

Swimming and swarming motilities were assayed to investigate whether the different concentrations of hydroquinine affect the motility of both DS and MDR *P. aeruginosa* strains. Hydroquinine had strong anti-motility effects in both swimming and swarming ability of both *P. aeruginosa* strains (Figure 5 and Figure 6). The control groups, including both strains untreated and with vehicle controls, showed normal swimming and swarming after 24 h incubation. No statistically significant difference was observed between the motility of the control groups. In contrast, treatment with hydroquinine significantly interfered with the swimming and swarming motilities in both DS and MDR *P. aeruginosa* strains in a dose-dependent manner. For the DS *P. aeruginosa* strain, the % swimming inhibitions by the hydroquinine concentrations of 2.500, 1.250, and 0.625 mg/mL were 50.0, 45.8, and 33.3%, respectively (Figure 5B), while the % swarming inhibitions by those were 54.0, 44.0, and 36.0% (*p* < 0.001), respectively (Figure 5D). For the MDR *P. aeruginosa* strain, the hydroquinine concentrations of 1.250, 0.625, and 0.312 mg/mL significantly inhibited swimming motility at 52.9, 35.2, and 23.5% inhibition, respectively (Figure 6B), whereas hydroquinine at only 1.250 mg/mL significantly inhibited swarming motility when compared to the controls (30.0%) (Figure 6D).

### 3.4. Inhibitory Effects of Hydroquinine on the Virulence Factor Productions and QS-Related Genes Were Exhibited in Both P. aeruginosa Strains

To investigate the effect of hydroquinine on QS-related genes and virulence factors in the *P. aeruginosa* strains, the relative expression of QS genes as well as production of pyocyanin and biofilm mass formation were determined. The QS-related genes, *rhlI* and *rhlR* in the DS and MDR *P. aeruginosa* strains were investigated using qRT-PCR technique after the 1 h treatment with 1.250 and 0.625 mg/mL of hydroquinine, respectively. The qRT-PCR results showed that the relative expression levels of the *rhlI* gene were significantly downregulated by 0.71 ± 0.07 and 0.37 ± 0.09-fold for the DS and MDR *P. aeruginosa* strains, respectively, while those of the *rhlR* gene were also significantly downregulated by 0.51 ± 0.17 and 0.50 ± 0.16-fold, respectively (Figure S1).

**Figure 7:**
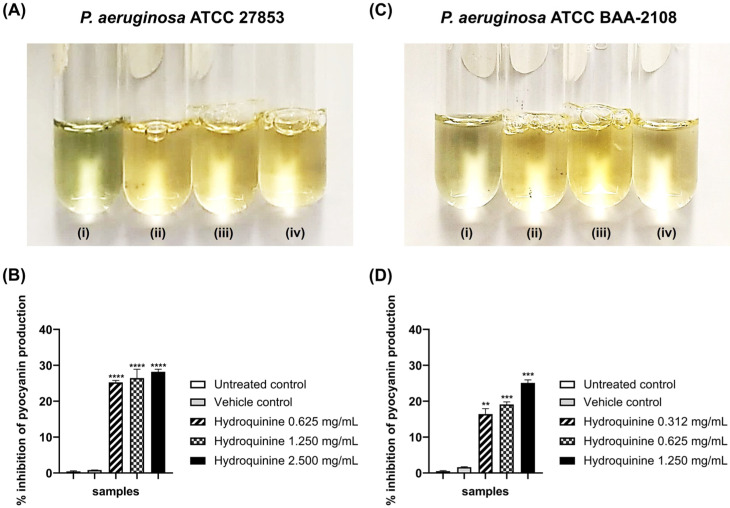
The effect of different concentrations of hydroquinine on pyocyanin production of (**A**,**B**) the DS *P. aeruginosa* ATCC 27853 and (**C**,**D**) the MDR *P. aeruginosa* ATCC BAA-2108 after 24 h at 35 ± 2 °C. For (**A**), (i) untreated control; (ii–iv) the hydroquinine concentrations at 0.625, 1.250 and 2.500 mg/mL, respectively. For (**C**), (i) untreated control; (ii–iv) the hydroquinine concentrations at 0.312, 0.625 and 1.250 mg/mL, respectively. The percentage inhibition of pyocyanin production in (**B**) the DS *P. aeruginosa* and (**D**) the MDR *P. aeruginosa* by the different concentrations of hydroquinine, compared with the control groups. Mean and standard deviation values from triplicate independent are shown. The asterisk **, *** and **** symbols are *p* < 0.01, *p* < 0.001 and *p* < 0.0001, respectively.

In addition, pyocyanin production (visualized as a green pigment) by both the DS and MDR *P. aeruginosa* strains were significantly reduced following treatment with hydroquinine, showing a dose-dependent response (Figure 7A,C). For the DS *P. aeruginosa* strain, the percentage inhibition of pyocyanin production by the hydroquinine concentrations at 2.500, 1.250, and 0.625 mg/mL for 24 h showed percentage inhibition of 27.13, 26.98, and 25.25%, respectively, when compared to the control groups (Figure 7B), whereas the MDR *P. aeruginosa* strain at 1.250, 0.625, and 0.312 mg/mL of hydroquinine for 24 h showed pyocyanin percentage inhibition of 25.12, 19.12, and 15.17%, respectively (Figure 7D).

**Figure 8:**
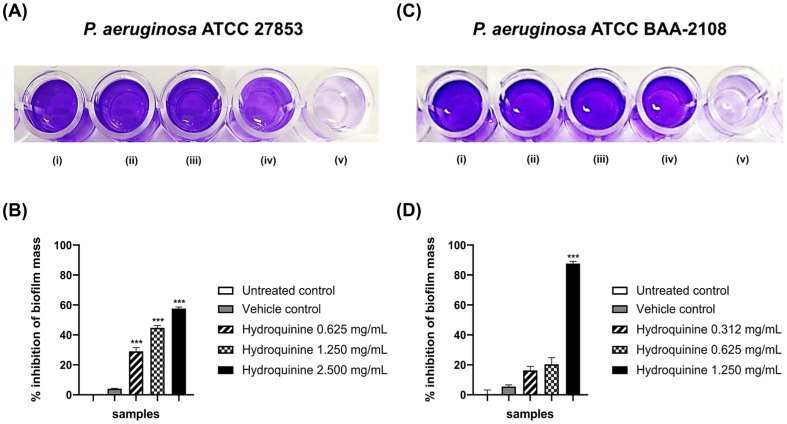
The effects of hydroquinine at different concentrations on biofilm formation. The panels (**A**) and (**B**) were the DS *P. aeruginosa* ATCC 27853 results, shown as (i) untreated control; (ii) vehicle control; and (iii–v) the hydroquinine concentrations at 0.625, 1.250 and 2.500 mg/mL, respectively, while the panels (**C**) and (**D**) were the MDR *P. aeruginosa* ATCC BAA-2108 results, shown as (i) untreated control; (ii) vehicle control; and (iii–v) the hydroquinine concentrations at 0.312, 0.625 and 1.250 mg/mL, respectively. The percentage inhibition of biofilm-forming ability in (**B**) the DS *P. aeruginosa* and (**D**) the MDR *P. aeruginosa* by the different concentrations of hydroquinine, compared with the control groups. Mean and standard deviation values from triplicate independent are shown. The asterisk *** symbol indicates *p* < 0.001.

The influence of hydroquinine on the biofilm-forming ability in both *P. aeruginosa* strains was investigated by measuring the optical density at 595 nm of biofilm-stained crystal violet after both *P. aeruginosa* strains treated with the different concentrations of hydroquinine at 35 ± 2 °C for 24 h. In the control groups, both *P. aeruginosa* strains showed normal biofilm formation. Hydroquinine reduced biofilm formation in both DS and MDR *P. aeruginosa* strains, compared to their controls (Figure 8). Specifically, against the MDR *P. aeruginosa* strain, hydroquinine (conc. 1.250 mg/mL) reduced biofilm mass formation by 87.65% (*p* < 0.0001). The sub-MIC concentrations of hydroquinine at 0.625 and 0.312 mg/mL also showed some inhibition of biofilm mass formation of the MDR *P. aeruginosa* strain, 20.40 and 16.30% respectively; however, this was not statistically significant (Figure 8D). In contrast, when *P. aeruginosa* ATCC 27853 was treated with hydroquinine at the concentrations of 2.500, 1.250 and 0.625 mg/mL, the biofilm mass formation was significantly inhibited by 57.61, 44.67 and 25.38%, respectively (Figure 8B).

## 4. Discussion

*P.* *aeruginosa*, a clinically opportunistic microorganism in hospitalized patients, can grow and produce virulence factors such as flagella, biofilms, pyocyanin, toxins, or degrading enzymes that harm human cells, resulting in increased virulence or hindering treatment [15,16,17,18]. In the present study, we investigated the anti-virulence factors of hydroquinine against both *P. aeruginosa* ATCC 27853 (DS) and ATCC BAA-2108 (MDR) strains. According to our recent group work [31], hydroquinine has antibacterial effects against both strains with the MIC values of 2.500 and 1.250 mg/mL, respectively. We also reported that hydroquinine could inhibit various pathogenic bacteria, e.g., *Staphylococcus aureus*, *Enterobacter cloacae*, *Escherichia coli*, and *Klebsiella pneumoniae*, with MICs ranging from 0.625 to 1.250 mg/mL [31]. 

Transcriptomic analysis was performed to determine the molecular responses in *P. aeruginosa* ATCC 27853 exposed to ½ × MIC (1.250 mg/mL) hydroquinine for 1 h. The 254 DEGs were identified hydroquinine-treated cells, compared to the untreated controls. Of the 254 DEGs, 157 genes were upregulated while 97 genes were downregulated. Using a combination GO enrichment annotation and KEGG analysis, our data suggested that hydroquinine downregulates genes involved in flagella formation, possibly affecting the motility function in *P. aeruginosa*. The flagellum is an important structure for the initial step of bacterial pathogenesis, enabling motility of the bacterium and allowing it to migrate, attach and colonize host cells as well as enabling its survival [39]. Moreover, flagella promote the uptake of essential nutrients, and as a result, has a critical role in the virulence of pathogenic organisms [40]. Transcriptomic analysis revealed that hydroquinine treatment of *P. aeruginosa* ATCC 27853 significantly downregulated 8 flagella-related genes, encoding the flagellar assembly of hook–filament junction (*flgK*), hook modification protein (*flgD*), basal body-associated proteins (*flgB*, *flgC*, *flgH*, *flgJ*, *fliF*) and motor-switch protein (*fliG*) (Table 2). Commonly, the basal body embedded in the cell membrane of Gram-negative bacteria, including *P. aeruginosa* (Figure 4), is the initial structure generated for flagellar assembly [41]. It is a rod structure with an inner ring (called as the membrane-supra-membrane ring; MS ring) and two outer rings (L- and P-rings) [42]. The MS ring is a supramolecular complex embedded in the cytoplasmic membrane, which consists of a single protein, FliF [41]. The FlgB and FlgC proteins are rod components assembled onto the MS ring to form the MS ring-rod complex [43]. The FlgJ, a distal rod cap protein, acts as a muramidase enzyme to degrade the peptidoglycan layers for constructing the rod structure in the periplasmic space, e.g., FlgG, FlgI and FlgH [43]. The presence of the L-ring (FlgH) connects with the outer membrane structure, whereas the P-ring interacts with the peptidoglycan layers [42]. In *P. aeruginosa*, the FliG protein is one of the flagellar cytoplasmic ring (C-ring) components [44], functioning as a flagellar motor switch [42]. The FlgK protein, a junction protein, links the hook/basal-body complex with the long flagellin filament [45]. The FlgD of *P. aeruginosa*, a flagella hook cap protein, plays an important role in the polymerization of the FlgE subunits to generate hook structure [45], which is essential for flagellar function [45,46]. The transcriptomic results, therefore, strongly suggest that hydroquinine causes the downregulation of the flagellar assembly genes, possibly affecting motility *P. aeruginosa*.

To validate the RNA sequencing findings, we examined whether hydroquinine affects the flagellar assembly genes in both DS and MDR *P. aeruginosa* strains by qRT-PCR. Three genes involved in generating the basal body (*flgH*, *flgC*, and *fliF*) and a hook–filament junction gene (*flgK*) were examined. Relative expression of all genes was downregulated in both DS and MDR *P. aeruginosa* strains after the ½MIC hydroquinine treatment for 1 h (Figure 5).

Phenotypically, swimming and swarming motilities of both DS and MDR *P. aeruginosa* were measured to investigate the anti-motility property of hydroquinine. The results showed that the swimming and swarming motilities were decreased following treatment with hydroquinine, concurrent with our genotypic analysis using high-throughput and qRT-PCR methods. In addition, the findings were also consistent with other studies on the anti-motility of other alkaloid compounds. For example, *P. aeruginosa* swarming motility is significantly inhibited by caffeine [25,27]. Other alkaloids (e.g., reserpine, piperine) inhibit both swimming and swarming motilities in *E.* coli by decreasing the expression levels of the flagellar gene (*fliC*) and motility genes (*motA* and *motB*) [47].

In *P. aeruginosa*, the QS system is essential for regulating the production of virulence factors, including pyocyanin and regulating biofilm formation [26,48]. Pyocyanin is zwitterionic, which can enter the host cytoplasmic membrane [49]. The presence of pyocyanin has a significant role in oxidative stress, leading to cytotoxicity in host cells [49]. Furthermore, the pyocyanin production of the bacterium is regulated by the *rhlI/R* QS system [50]. To examine whether hydroquinine affects pyocyanin production via the QS-dependent system, pyocyanin production as well as *rhlI* and *rhlR* expression were investigated in both DS and MDR *P. aeruginosa* strains following hydroquinine treatment. It was found that hydroquinine significantly inhibited the pyocyanin production and the mRNA expression levels of the rhl QS system (*rhlI* and *rhlR*) in both strains, suggesting that hydroquinine could reduce pyocyanin production by inhibiting the *rhl* QS system. This was consistent with reports from Park et al. (2008) [26] that the *rhlI*/*R* QS system is related to pyocyanin production. It has been demonstrated that the absence of C4-HSL, a known autoinducer for activating the *rhl* QS system, decreases pyocyanin production in *P. aeruginosa*. In addition, other alkaloids (e.g., solenopsin A) and derivative compounds presented anti-QS activity and anti-pyocyanin production in *P. aeruginosa* by inhibiting the production of QS molecules [25,26,27].

Finally, the biofilm-forming ability was also investigated after treatment with hydroquinine. We show that at sub-MICs of hydroquinine, biofilm mass in *P. aeruginosa* is reduced (Figure 8). This may occur through repression of the *rhl* QS system or by the disruption of flagellar motility. Previous reports have shown that the *rhlI* mutant or null *rhl* QS genes showed the reduction of biofilm mass, compared with the wild-type strain [51]. Moreover, biofilm-forming capacity in *P. aeruginosa* is related to the flagella motility, which is an early stage in attachment for colonization and biofilm formation [41,52]. Similar to other alkaloid derivative compounds, biofilm formation was inhibited in *P. aeruginosa* [22,24,27]. The findings suggest that hydroquinine has potential as an anti-biofilm formation agent. Since biofilm-forming *P. aeruginosa* is less susceptible to antibiotics, this makes it difficult to eliminate them completely [53]; therefore, hydroquinine may be used as an alternative, in combination with other antibiotics. However, further investigation is still required.

## 5. Conclusions

In this study, high-throughput transcriptomic analysis was used to investigate the molecular responses of *P. aeruginosa* to hydroquinine. The transcriptomic profiles revealed that hydroquinine significantly altered the expression levels of the 254 transcripts in *P. aeruginosa* ATCC 27853, including the upregulation of 97 transcripts and the downregulation of 157 transcripts. GO enrichment analysis of downregulated genes suggested that hydroquinine interfered with flagellar assembly and motility functions in both *P. aeruginosa* ATCC 27853 (DS) and ATCC BAA-2108 (MDR) strains. Furthermore, hydroquinine decreased both swimming and swarming motility functions in *P. aeruginosa*. Moreover, hydroquinine also exhibited anti-pyocyanin properties by downregulating the QS molecules (*rhlI* and *rhlR*). To conclude, these findings demonstrated that hydroquinine has anti-virulence factor properties in both DS and MDR *P. aeruginosa* strains. As a result, hydroquinine has the potential to be used as anti-infective and anti-virulence factor agents in clinical settings, especially in *P. aeruginosa* cases. Furthermore, this potential mechanism of action may help to explain how hydroquinine has bacterial inhibitory activity against both drug-sensitive and multidrug-resistant strains of *P. aeruginosa* cases. Further elucidation of the inhibitory mechanisms of hydroquinine against *P. aeruginosa* should be investigated in the future to determine its potential use in the clinic.

## Figures and Tables

**Table 1:** Primer sequences and annealing temperature were used in this study.

Primer Name	Oligonucleotide Sequences (5′ to 3′)	Annealing Temperature (°C)	References
*flgH F*	CGAGCAGAACCTCTACGACG	57.5	This study
*flgH R*	TCGGGTTGTTGGTGGTCATG	57.5	This study
*flgK F*	CCAGCAAGCTGAATTCCAGC	56.0	This study
*flgK R*	GGTCGTCTCGATATCGCTGG	56.0	This study
*fliF F*	AGATGTACAACCCGGACCAG	57.5	This study
*fliF R*	TCGGATCGATGATGGTCTGG	57.5	This study
*flgC F*	TTCTCCACCATGTTCCAGCAG	57.5	This study
*flgC R*	TCCTCGACCACGTTCACATTG	57.5	This study
*rhlI F*	CAGGAATTCGACCAGTTCGACC	58.5	This study
*rhlI R*	CGAAGACGTCCTTGAGCAGG	58.5	This study
*rhlR F*	GTAGCGAGATGCAGCCGATC	57.0	This study
*rhlR R*	CCTTGGGATAGGTGCCATGG	57.0	This study
*16s rRNA F*	CATGGCTCAGATTGAACGCTG	58.0	[31]
*16s rRNA R*	GCTAATCCGACCTAGGCTCATC	58.0	[31]

**Table 2:** Differentially expressed genes (DEGs) associated with the flagellar assembly as determined by transcriptome analysis.

Gene Name	Gene Product	Log_2_ FC ^1^	FDR ^2^	*p* Value
*flgC*	Flagellar basal-body rod protein FlgC	−2.93	3.90 × 10^−3^	6.66 × 10^−5^
*flgB*	Flagellar basal-body rod protein FlgB	−2.38	2.43 × 10^−2^	8.00 × 10^−4^
*flgJ*	Flagellar protein FlgJ	−2.33	2.41 × 10^−2^	8.00 × 10^−4^
*flgD*	Flagellar basal-body rod modification protein FlgD	−2.33	2.76 × 10^−2^	1.00 × 10^−3^
*fliF*	Flagellar M-ring outer membrane protein precursor FliF	−2.26	2.51 × 10^−2^	1.00 × 10^−3^
*fliG*	Flagellar motor-switch protein 1 FliG	−2.23	2.29 × 10^−2^	1.20 × 10^−3^
*flgK*	Flagellar hook-associated protein 1 FlgK	−2.23	3.22 × 10^−2^	1.50 × 10^−3^
*flgH*	Flagellar L-ring protein precursor FlgH	−2.18	4.13 × 10^−2^	1.80 × 10^−3^

^1^ Log_2_ FC, Log_2_ relative fold changes in expressed genes in response to hydroquinine, compared to the untreated control. ^2^ FDR, false discovery rate with a statistical significance of *p* value ≤ 0.05.

## Data Availability

Not applicable.

## Supplementary Materials

The following supporting information can be downloaded at: https://www.mdpi.com/article/10.3390/antibiotics11101436/s1, Table S1: lists of downregulated and upregulated DEGs. Figure S1. The relative expression of quorum sensing-related genes in (A) *P. aeruginosa* ATCC 27853 strain with hydroquinine at 1.250 mg/mL for 1 h and (B) *P. aeruginosa* ATCC BAA-2108 strain treated with hydroquinine at 0.625 mg/mL for 1 h, compared to the corresponding untreated control. The asterisk ** symbol was *p* < 0.01.
