# Supplementary material for: High-Throughput Transcriptomic Profiling Reveals the Inhibitory Effect of Hydroquinine on Virulence Factors in Pseudomonas aeruginosa
Source: Antibiotics (Basel). 2022 Oct 19;11(10):1436. doi: 10.3390/antibiotics11101436 (PMC9598861; doi:10.3390/antibiotics11101436)
Supplement: Supplementary file 1 [file antibiotics-11-01436-s001.zip › antibiotics-1967808-Supplementary.pdf]

Table S1. Significant differential expression genes (DEGs) of *P. aeruginosa* ATCC 27853 treated with 1.250 mg/mL of hydroquinine

DEGs	Log_2FC	logCPM	*P*-Value	FDR	Product	RefSeq
PA4599	9.47	11.10	4.79E-23	2.56E-19	Resistance-Nodulation-Cell Division (RND) multidrug efflux membrane fusion protein MexC precursor	NP_253289
PA1225	7.93	10.12	9.46E-19	2.53E-15	NAD(P)H dehydrogenase	NP_249916
PA4990	6.84	8.40	3.91E-15	5.22E-12	SMR multidrug efflux transporter	NP_253677
PA2932	6.69	9.02	3.85E-15	5.22E-12	morphinone reductase	NP_251622
PA4598	6.27	9.81	3.59E-14	3.83E-11	Resistance-Nodulation-Cell Division (RND) multidrug efflux transporter MexD	NP_253288
PA4354	6.10	10.73	8.44E-14	7.51E-11	hypothetical protein	NP_253044
PA1282	6.05	7.83	5.86E-13	3.91E-10	major facilitator superfamily (MFS) transporter	NP_249973
PA4597	6.02	8.32	3.80E-13	2.90E-10	Multidrug efflux outer membrane protein OprJ precursor	NP_253287
PA3720	5.83	7.55	2.79E-12	1.66E-09	hypothetical protein	NP_252409
PA3719	5.71	5.60	1.48E-10	6.10E-08	antirepressor for MexR, ArmR	NP_252408
PA2931	5.31	9.57	1.72E-11	9.16E-09	CifR	NP_251621
PA0465	5.29	8.12	3.57E-11	1.73E-08	inner membrane protein CreD	NP_249156
PA2019	5.26	7.85	5.55E-11	2.47E-08	Resistance-Nodulation-Cell Division (RND) multidrug efflux membrane fusion protein MexX precursor	NP_250709
PA2850	5.24	6.77	1.78E-10	6.77E-08	organic hydroperoxide resistance protein	NP_251540
PA0466	4.95	5.27	6.64E-09	1.48E-06	hypothetical protein	NP_249157
PA4288	4.90	7.31	6.93E-10	1.85E-07	transcriptional regulator	NP_252978
PA2018	4.90	8.95	2.88E-10	9.60E-08	Resistance-Nodulation-Cell Division (RND) multidrug efflux transporter MexY	NP_250708
PA0534	4.89	8.64	3.31E-10	1.04E-07	FAD-dependent oxidoreductase	NP_249225
PA4623	4.88	10.51	2.29E-10	8.16E-08	hypothetical protein	NP_253313
PA3126	4.82	10.69	3.52E-10	1.04E-07	heat-shock protein IbpA	NP_251816
PA3690	4.75	9.98	5.84E-10	1.64E-07	metal-transporting P-type ATPase	NP_252380
PA1297	4.56	7.14	5.13E-09	1.19E-06	metal transporter	NP_249988
PA1283	4.56	7.50	3.84E-09	9.32E-07	transcriptional regulator	NP_249974
PA4881	4.51	11.57	2.26E-09	5.75E-07	hypothetical protein	NP_253568
PA0125	4.42	7.31	9.55E-09	2.04E-06	ParD antitoxin	NP_248815
PA1970	4.29	7.78	1.66E-08	3.16E-06	hypothetical protein	NP_250660

PA4596	4.28	6.35	4.18E-08	6.98E-06	EsrC	NP_253286
PA2274	4.24	3.65	4.90E-06	0.0005	hypothetical protein	NP_250964
PA3732	4.22	9.97	1.56E-08	3.10E-06	Uncharacterized protein	NP_252421
PA3731	4.19	10.15	1.92E-08	3.53E-06	hypothetical protein	NP_252420
PA0124	4.19	7.31	3.44E-08	6.12E-06	ParE toxin	NP_248814
PA1290	4.12	5.46	2.95E-07	3.75E-05	transcriptional regulator	NP_249981
PA4355	4.10	9.36	3.61E-08	6.22E-06	PyeM	NP_253045
PA0736a	4.06	7.58	6.95E-08	1.09E-05	hypothetical protein	NP_249427
PA1744	4.03	6.40	1.42E-07	2.06E-05	hypothetical protein	NP_250435
PA0737	3.98	6.98	1.36E-07	2.06E-05	hypothetical protein	NP_249428
PA1298	3.96	5.55	5.19E-07	6.30E-05	hypothetical protein	NP_249989
PA1223	3.95	6.41	2.71E-07	3.61E-05	transcriptional regulator	NP_249914
PA1137	3.84	8.25	2.11E-07	2.88E-05	oxidoreductase	NP_249828
PA0474	3.71	2.81	0.0002	0.0096	hypothetical protein	NP_249165
PA1503	3.67	2.78	0.0002	0.0104	hypothetical protein	NP_250194
PA1030	3.67	9.40	4.81E-07	5.97E-05	hypothetical protein	NP_249721
PA2782	3.61	2.23	0.001141	0.0306	biofilm-associated metzincin Inhibitor, BamI	NP_251472
PA2277	3.59	5.60	2.88E-06	0.0003	ArsR protein	NP_250967
PA2054	3.53	5.30	5.05E-06	0.0005	transcriptional regulator CynR	NP_250744
PA2433	3.50	4.98	8.90E-06	0.0007	hypothetical protein	NP_251123
PA1942	3.49	8.82	1.44E-06	0.0002	hypothetical protein	NP_250632
PA1291	3.47	6.42	2.94E-06	0.0003	hypothetical protein	NP_249982
PA3390	3.41	4.40	3.08E-05	0.0022	hypothetical protein	NP_252079
PA3062	3.40	6.23	5.02E-06	0.0005	PelC	NP_251752
PA4986	3.38	7.37	3.47E-06	0.0004	oxidoreductase	NP_253673
PA1343	3.33	8.47	3.81E-06	0.0004	hypothetical protein	NP_250034
PA0424	3.32	6.87	5.47E-06	0.0005	multidrug resistance operon repressor MexR	NP_249115
PA2469	3.28	4.70	3.35E-05	0.0023	transcriptional regulator	NP_251159
PA0476	3.28	3.56	0.000127	0.0065	permease	NP_249167
PA2746a	3.27	6.30	8.97E-06	0.0007	hypothetical protein	NP_251436
PA2933	3.27	3.89	7.76E-05	0.0044	major facilitator superfamily (MFS) transporter	NP_251623
PA3962	3.24	7.94	6.96E-06	0.0006	hypothetical protein	NP_252651
PA4762	3.23	10.53	5.87E-06	0.0005	heat shock protein GrpE	NP_253450
PA4600	3.21	8.02	7.62E-06	0.0007	transcriptional regulator NfxB	NP_253290
PA4985	3.19	6.79	1.13E-05	0.0009	Uncharacterized protein	NP_253672
PA1743	3.19	5.67	2.22E-05	0.0016	hypothetical protein	NP_250434
PA3819	3.18	10.80	7.70E-06	0.0007	hypothetical protein	NP_252508
PA1316	3.17	5.11	3.23E-05	0.0023	major facilitator superfamily (MFS) transporter	NP_250007
PA1962	3.16	3.46	0.000233	0.0102	FMN-dependent NADH-azoreductase 2, AzoR2	NP_250652
PA1066	3.08	5.73	3.48E-05	0.0023	short-chain dehydrogenase	NP_249757
PA3133	3.07	5.53	3.58E-05	0.0024	SawR	NP_251823
PA1344	3.05	8.52	1.74E-05	0.0013	short-chain dehydrogenase	NP_250035

PA1596	3.04	10.72	1.65E-05	0.0012	heat shock protein HtpG	NP_250287
PA2109	3.03	3.35	0.000398	0.0146	hypothetical protein	NP_250799
PA4902	3.02	5.86	4.25E-05	0.0027	transcriptional regulator	NP_253589
PA5382	3.00	6.12	3.80E-05	0.0025	transcriptional regulator	NP_254069
PA0779	2.93	10.57	3.04E-05	0.0022	AsrA	NP_249470
PA3552	2.90	7.60	4.38E-05	0.0028	ArnB	NP_252242
PA4495	2.89	9.15	4.04E-05	0.0026	hypothetical protein	NP_253185
PA3718	2.89	4.06	0.000365	0.0138	major facilitator superfamily (MFS) transporter	NP_252407
PA3554	2.86	8.43	4.96E-05	0.0031	ArnA	NP_252244
PA0123	2.83	6.56	7.91E-05	0.0044	transcriptional regulator	NP_248813
PA3064	2.79	7.83	7.46E-05	0.0042	PelA	NP_251754
PA3952	2.79	7.04	8.96E-05	0.0049	hypothetical protein	NP_252641
PA0942	2.75	6.94	0.0001	0.0058	transcriptional regulator	NP_249633
PA0738	2.74	3.94	0.0007	0.0226	hypothetical protein	NP_249429
PA3132	2.73	5.36	0.0002	0.0099	hydrolase	NP_251822
PA1507	2.72	5.09	0.0002	0.0102	probable transporter	NP_250198
PA4761	2.71	12.24	0.0001	0.0053	DnaK protein	NP_253449
PA2930	2.70	5.68	0.0002	0.0094	transcriptional regulator	NP_251620
PA5053	2.70	9.33	0.0001	0.0057	heat shock protein HslV	NP_253740
PA3597	2.70	3.41	0.0011	0.0299	amino acid permease	NP_252287
PA1471	2.69	5.73	0.0002	0.0094	hypothetical protein	NP_250162
PA2938	2.68	4.18	0.0007	0.0216	probable transporter	NP_251628
PA2009	2.67	7.09	0.0002	0.0077	homogentisate 1,2-dioxygenase	NP_250699
PA2815	2.64	9.96	0.0001	0.0072	acyl-CoA dehydrogenase	NP_251505
PA2246	2.62	4.36	0.0007	0.0225	transcriptional regulator BkdR	NP_250936
PA4773	2.61	5.33	0.0004	0.0146	SpeD2	NP_253461
PA0907	2.60	6.10	0.0003	0.0118	lysis phenotype activator, AlpA	NP_249598
PA1541	2.58	7.43	0.0002	0.0102	probable drug efflux transporter	NP_250232
PA0604	2.58	5.44	0.0004	0.0152	AgtB	NP_249295
PA1921	2.56	3.56	0.0023	0.0482	hypothetical protein	NP_250611
PA2776	2.56	8.75	0.0002	0.0102	FAD-dependent oxidoreductase	NP_251466
PA3063	2.55	7.83	0.0003	0.0108	PelB	NP_251753
PA0740	2.53	3.27	0.0023	0.0483	SDS hydrolase SdsA1	NP_249431
PA2315	2.53	3.27	0.0023	0.0483	hypothetical protein	NP_251005
PA1852	2.53	5.47	0.0005	0.0170	hypothetical protein	NP_250543
PA3729	2.50	8.55	0.0003	0.0127	hypothetical protein	NP_252418
PA0037	2.50	6.30	0.0004	0.0151	transcriptional regulator TrpI	NP_248727
PA3061	2.49	6.90	0.0004	0.0146	PelD	NP_253315
PA4625	2.47	9.95	0.0003	0.0132	cyclic diguanylate-regulated TPS partner A, CdrA	NP_253315
PA1592	2.47	9.89	0.0003	0.0133	hypothetical protein	NP_250283
PA3730	2.46	6.72	0.0005	0.0167	hypothetical protein	NP_252419

PA2849	2.46	6.15	0.0005	0.0180	OhrR	NP_251539
PA4386	2.45	10.07	0.0004	0.0142	GroES protein	NP_253076
PA4710	2.45	6.79	0.0005	0.0167	Heme/Hemoglobin uptake outer membrane receptor PhuR precursor	NP_253398
PA1502	2.45	4.29	0.0014	0.0362	glyoxylate carboligase	NP_250193
PA5428	2.45	6.67	0.0005	0.0167	transcriptional regulator	NP_254115
PA5212	2.44	8.91	0.0004	0.0152	hypothetical protein	NP_253899
PA0794	2.41	8.90	0.0005	0.0167	aconitate hydratase	NP_249485
PA2441	2.40	6.52	0.0006	0.0210	hypothetical protein	NP_251131
PA3672	2.40	6.33	0.0007	0.0219	probable ATP-binding component of ABC transporter	NP_252362
PA1571	2.40	4.45	0.0015	0.0367	hypothetical protein	NP_250262
PA1229	2.40	4.56	0.0014	0.0345	transcriptional regulator	NP_249920
PA2478	2.37	5.55	0.0009	0.0265	thiol:disulfide interchange protein	NP_251168
PA1542	2.37	6.31	0.0008	0.0235	hypothetical protein	NP_250233
PA2020	2.37	6.73	0.0007	0.0226	MexZ	NP_250710
PA1226	2.37	6.54	0.0007	0.0226	transcriptional regulator	NP_249917
PA2886	2.36	5.26	0.0012	0.0311	expressed protein with apparent function in citronellol catabolism	NP_251576
PA3559	2.35	6.82	0.0008	0.0234	nucleotide sugar dehydrogenase	NP_252249
PA3553	2.35	7.23	0.0007	0.0226	ArnC	NP_252243
PA0922	2.34	6.83	0.0008	0.0238	hypothetical protein	NP_249613
PA4675	2.33	8.24	0.0007	0.0226	ChtA	NP_253364
PA5178	2.31	9.24	0.0008	0.0235	hypothetical protein	NP_253865
PA3370	2.29	6.16	0.0011	0.0305	hypothetical protein	NP_252060
PA0763	2.28	11.05	0.0009	0.0257	anti-sigma factor MucA	NP_249454
PA3059	2.27	7.05	0.0011	0.0293	PelF	NP_251749
PA2322	2.25	5.33	0.0017	0.0411	GntP	NP_251012
PA1631	2.24	5.26	0.0020	0.0442	acyl-CoA dehydrogenase	NP_250322
PA2510	2.23	5.25	0.0020	0.0448	transcriptional regulator CatR	NP_251200
PA1178	2.23	11.91	0.0011	0.0306	PhoP/Q and low Mg2+ inducible outer membrane protein H1 precursor	NP_249869
PA4385	2.22	12.54	0.0011	0.0306	GroEL protein	NP_253075
PA3007	2.22	8.97	0.0012	0.0322	repressor protein LexA	NP_251697
PA3670	2.20	7.03	0.0015	0.0377	hypothetical protein	NP_252360
PA0833	2.20	10.34	0.0013	0.0337	hypothetical protein	NP_249524
PA5473	2.20	8.82	0.0014	0.0345	hypothetical protein	NP_254160
PA0119	2.19	6.06	0.0018	0.0420	dicarboxylate transporter	NP_248809
PA2493	2.19	9.75	0.0014	0.0345	Resistance-Nodulation-Cell Division (RND) multidrug efflux membrane fusion protein MexE precursor	NP_251183

PA3369	2.19	6.61	0.0017	0.0405	hypothetical protein	NP_252059
PA2111	2.17	5.43	0.0024	0.0500	hypothetical protein	NP_250801
PA1332	2.16	6.96	0.0018	0.0416	hypothetical protein	NP_250023
PA3058	2.15	6.14	0.0022	0.0480	PelG	NP_251748
PA1597	2.15	6.98	0.0020	0.0442	hypothetical protein	NP_250288
PA3728	2.14	8.36	0.0018	0.0413	hypothetical protein	NP_252417
PA2483	2.14	7.38	0.0019	0.0441	hypothetical protein	NP_251173
PA1016	2.14	5.98	0.0023	0.0492	hypothetical protein	NP_249707
PA0754	2.14	6.38	0.0022	0.0470	hypothetical protein	NP_249445
PA4581	2.13	7.05	0.0021	0.0458	transcriptional regulator RtcR	NP_253271
PA2830	2.10	9.66	0.0020	0.0450	heat shock protein HtpX	NP_251520
PA4876	2.10	8.12	0.0022	0.0470	osmotically inducible lipoprotein OsmE	NP_253563
PA5054	2.09	9.89	0.0021	0.0468	heat shock protein HslU	NP_253741
PA1457	-2.09	8.31	0.0022	0.0480	chemotaxis protein CheZ	NP_250148
PA1561	-2.11	9.31	0.0019	0.0441	aerotaxis receptor Aer	NP_250252
PA5030	-2.14	7.35	0.0019	0.0432	major facilitator superfamily (MFS) transporter	NP_253717
PA5027	-2.15	8.49	0.0017	0.0405	hypothetical protein	NP_253714
PA1456	-2.16	7.17	0.0018	0.0413	two-component response regulator CheY	NP_250147
PA4921	-2.16	6.21	0.0020	0.0448	cholinesterase, ChoE	NP_253608
PA3912	-2.17	6.62	0.0019	0.0431	hypothetical protein	NP_252601
PA5207	-2.17	7.44	0.0017	0.0405	phosphate transporter	NP_253894
PA3613	-2.17	10.21	0.0015	0.0367	hypothetical protein	NP_252303
PA1083	-2.18	6.55	0.0018	0.0413	flagellar L-ring protein precursor FlgH	NP_249774
PA0518	-2.19	7.93	0.0015	0.0364	cytochrome c-551 precursor	NP_249209
PA4523	-2.20	7.80	0.0014	0.0356	hypothetical protein	NP_253213
PA3350	-2.20	5.71	0.0019	0.0431	hypothetical protein	NP_252040
PA0519	-2.21	9.86	0.0013	0.0328	nitrite reductase precursor	NP_249210
PA1086	-2.21	6.50	0.0015	0.0370	flagellar hook-associated protein 1 FlgK	NP_249777
PA0526	-2.22	5.89	0.0017	0.0405	hypothetical protein	NP_249217
PA5159	-2.22	5.27	0.0020	0.0442	multidrug resistance protein	NP_253846
PA1102	-2.23	7.56	0.0012	0.0322	flagellar motor switch protein FliG	NP_249793
PA4348	-2.24	9.12	0.0011	0.0305	hypothetical protein	NP_253038
PA4074	-2.25	5.76	0.0015	0.0367	transcriptional regulator	NP_252763
PA3429	-2.26	4.98	0.0018	0.0425	epoxide hydrolase	NP_252119
PA1101	-2.26	7.63	0.0010	0.0292	Flagella M-ring outer membrane protein precursor	NP_249792
PA4551	-2.27	5.66	0.0014	0.0355	type 4 fimbrial biogenesis protein PilV	NP_253241
PA4796	-2.28	4.96	0.0017	0.0405	hypothetical protein	NP_253484
PA4535	-2.29	5.72	0.0013	0.0328	hypothetical protein	NP_253225
PA4536	-2.31	5.50	0.0013	0.0345	hypothetical protein	NP_253226

PA3394	-2.31	6.23	0.0010	0.0283	NosF protein	NP_252084
PA3913	-2.32	6.99	0.0009	0.0258	protease	NP_252602
PA1085	-2.33	6.83	0.0008	0.0241	flagellar protein FlgJ	NP_249776
PA1079	-2.33	6.09	0.0010	0.0276	flagellar basal-body rod modification protein FlgD	NP_249770
PA4359	-2.33	5.55	0.0011	0.0306	hypothetical protein	NP_253049
PA1555	-2.34	10.66	0.0007	0.0216	Cytochrome c oxidase, cbb3-type, CcoP subunit	NP_250246
PA2119	-2.35	8.64	0.0007	0.0216	alcohol dehydrogenase (Zn-dependent)	NP_250809
PA3432	-2.35	5.82	0.0009	0.0265	hypothetical protein	NP_252122
PA1736	-2.36	7.02	0.0007	0.0226	acyl-CoA thiolase	NP_250427
PA1557	-2.37	11.39	0.0006	0.0187	Cytochrome c oxidase, cbb3-type, CcoN subunit	NP_250248
PA1077	-2.38	5.73	0.0008	0.0243	flagellar basal-body rod protein FlgB	NP_249768
PA4550	-2.39	5.69	0.0009	0.0257	type 4 fimbrial biogenesis protein FimU	NP_253240
PA5497	-2.43	10.07	0.0004	0.0152	class II (cobalamin-dependent) ribonucleotide-diphosphate reductase subunit, NrdJa	NP_254184
PA4100	-2.43	9.26	0.0004	0.0154	dehydrogenase	NP_252789
PA0951a	-2.44	5.69	0.0007	0.0219	ribonuclease	NP_249642
PA4517	-2.46	5.19	0.0007	0.0226	chemotaxis protein CheZ	NP_250148
PA1103	-2.46	6.84	0.0005	0.0157	flagellar assembly protein	NP_249794
PA5160	-2.46	5.01	0.0007	0.0226	drug efflux transporter	NP_253847
PA2126	-2.48	6.53	0.0005	0.0157	cupA gene regulator C, CgrC	NP_250816
PA1546	-2.48	10.22	0.0003	0.0130	oxygen-independent coproporphyrinogen III oxidase	NP_250237
PA1555	-2.48	8.74	0.0003	0.0131	Cytochrome c oxidase, cbb3-type, CcoP subunit	NP_250246
PA4465	-2.48	9.36	0.0003	0.0130	hypothetical protein	NP_253155
PA4571	-2.49	8.95	0.0003	0.0128	cytochrome c	NP_253261
PA4328	-2.49	8.07	0.0003	0.0130	hypothetical protein	NP_253018
PA5496	-2.50	7.91	0.0003	0.0130	class II (cobalamin-dependent) ribonucleotide-diphosphate reductase subunit, NrdJb	NP_254183
PA0141	-2.51	9.78	0.0003	0.0117	hypothetical protein	NP_248831
PA3431	-2.52	6.13	0.0004	0.0152	hypothetical protein	NP_252121
PA4587	-2.52	10.39	0.0003	0.0109	cytochrome c551 peroxidase precursor	NP_253277
PA4073	-2.58	10.37	0.0002	0.0093	aldehyde dehydrogenase	NP_252762
PA1556	-2.59	10.05	0.0002	0.0087	Cytochrome c oxidase, cbb3-type, CcoO subunit	NP_250247
PA3416	-2.60	3.58	0.0011	0.0306	pyruvate dehydrogenase E1 component, beta chain	NP_252106

PA0527	-2.61	7.55	0.0002	0.0091	transcriptional regulator Dnr	NP_249218
PA3614	-2.62	8.96	0.0002	0.0081	hypothetical protein	NP_252304
PA0517	-2.63	7.31	0.0002	0.0087	c-type cytochrome precursor nirC	NP_249208
PA2380	-2.64	3.82	0.0009	0.0252	hypothetical protein	NP_251070
PA3049	-2.64	7.63	0.0002	0.0081	ribosome modulation factor	NP_251739
**PA0515**	**-2.66**	**7.25**	**0.0002**	**0.0077**	**transcriptional regulator**	**NP_249206**
PA3919	-2.68	9.36	0.0001	0.0062	hypothetical protein	NP_252608
PA2567	-2.72	8.51	0.0001	0.0053	hypothetical protein	NP_251257
PA5026	-2.74	5.11	0.0002	0.0104	hypothetical protein	NP_253713
PA2118a	-2.74	5.07	0.0002	0.0104	O6-methylguanine-DNA methyltransferase	NP_250808
PA0512	-2.78	6.59	9.55E-05	0.0051	NirH	NP_249203
PA0178	-2.79	4.82	0.0002	0.0098	two-component sensor	NP_248868
PA5475	-2.83	9.45	5.45E-05	0.0032	hypothetical protein	NP_254162
PA1920	-2.85	8.95	5.15E-05	0.0031	class III (anaerobic) ribonucleoside-triphosphate reductase subunit, NrdD	NP_250610
PA3458	-2.88	7.51	5.16E-05	0.0031	transcriptional regulator	NP_252148
PA0276	-2.89	5.48	8.29E-05	0.0046	hypothetical protein	NP_248967
PA4071	-2.91	6.63	5.33E-05	0.0032	hypothetical protein	NP_252760
PA1918	-2.91	4.56	0.000149	0.0074	hypothetical protein	NP_250608
PA1078	-2.93	5.59	6.66E-05	0.0039	flagellar basal-body rod protein FlgC	NP_249769
PA0516	-2.94	8.52	3.29E-05	0.0023	heme d1 biosynthesis protein NirF	NP_249207
PA0514	-2.97	6.74	3.48E-05	0.0023	heme d1 biosynthesis protein NirL	NP_249205
PA1916	-3.01	3.19	0.000785	0.0235	amino acid permease	NP_250606
PA4072	-3.06	9.02	1.59E-05	0.0012	amino acid permease	NP_252761
PA2318	-3.06	9.22	1.54E-05	0.0012	hypothetical protein	NP_251008
PA0513	-3.15	6.59	1.49E-05	0.0012	NirG	NP_249204
PA3337	-3.16	9.34	8.99E-06	0.0007	ADP-L-glycero-D-mannoheptose 6-epimerase	NP_252027
PA5231	-3.17	9.69	8.33E-06	0.0007	ATP-binding/permease fusion ABC transporter	NP_253918
PA0714	-3.17	4.38	5.59E-05	0.0033	hypothetical protein	NP_249405
PA2317	-3.29	9.54	4.23E-06	0.0004	oxidoreductase	NP_251007
PA5172	-3.32	12.87	3.36E-06	0.0004	ornithine carbamoyltransferase, catabolic	NP_253859
PA4681	-3.35	3.97	7.15E-05	0.0041	hypothetical protein	NP_253370
PA5173	-3.41	12.45	1.96E-06	0.0002	carbamate kinase	NP_253860
PA5230	-3.54	8.10	1.23E-06	0.0001	permease of ABC transporter	NP_253917
PA1917	-3.56	2.04	0.002228	0.0480	hypothetical protein	NP_250607
PA5171	-3.85	13.39	1.43E-07	2.06E-05	arginine deiminase	NP_253858
PA1919	-3.94	7.41	1.49E-07	2.09E-05	class III (anaerobic) ribonucleoside-triphosphate	NP_250609

					reductase activating protein, 'activase', NrdG	
PA4682	-3.98	5.20	5.82E-07	6.90E-05	hypothetical protein	NP_253371
PA5170	-4.24	12.72	1.22E-08	2.51E-06	arginine/ornithine antiporter	NP_253857
PA4683	-4.25	6.35	5.28E-08	8.54E-06	hypothetical protein	NP_253372
PA0713	-5.07	3.90	2.81E-07	3.66E-05	hypothetical protein	NP_249404

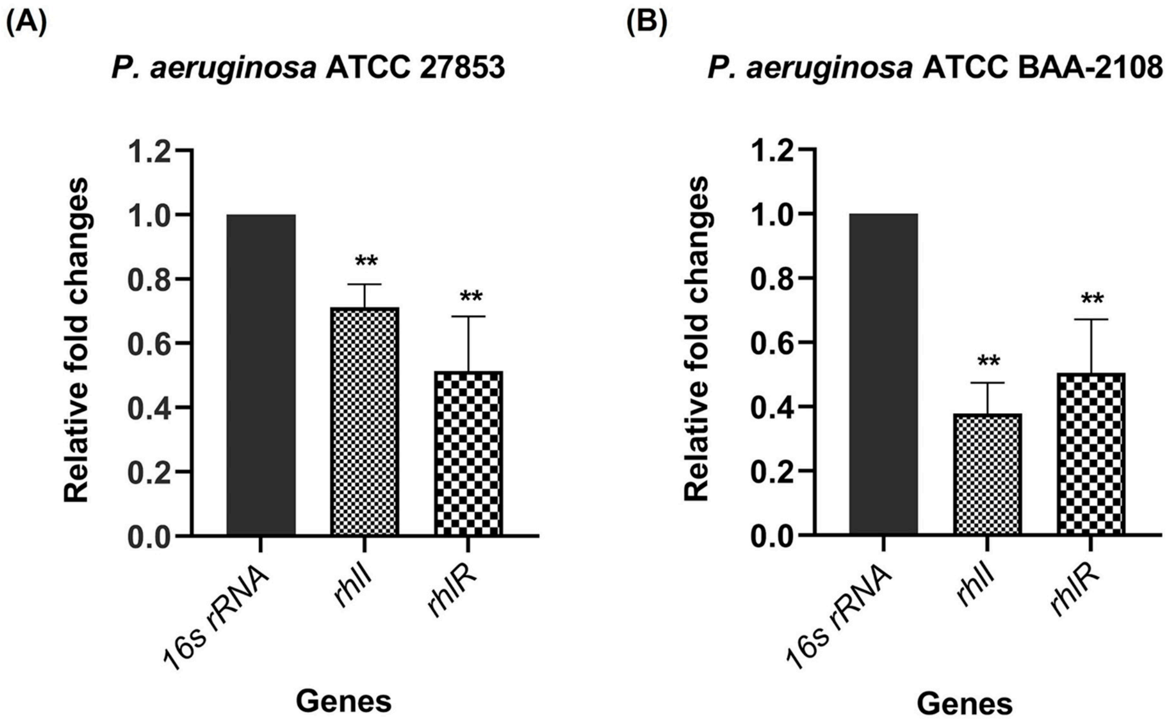

Figure S1. The relative expression of quorum sensing-related genes in **(A)** *P. aeruginosa* ATCC 27853 strain with hydroquinine at 1.250 mg/mL for 1 h and **(B)** *P. aeruginosa* ATCC BAA-2108 strain treated with hydroquinine at 0.625 mg/mL for 1 h, compared to the corresponding untreated control. The asterisk ** symbol was $p < 0.01$.
